# Effectiveness and Cost of Using Facebook Recruitment to Elicit Canadian Women’s Perspectives on Bone Health and Osteoporosis: Cross-Sectional Survey Study

**DOI:** 10.2196/47970

**Published:** 2023-09-29

**Authors:** Emma Olive Billington, Charley M Hasselaar, Lorena Kembel, Rebecca C Myagishima, Mubashir A Arain

**Affiliations:** 1 Cumming School of Medicine University of Calgary Calgary, AB Canada; 2 McCaig Institute for Bone & Joint Health Calgary, AB Canada; 3 Dr David Hanley Osteoporosis Centre Alberta Health Services Calgary, AB Canada; 4 Health Systems Evaluation & Evidence Alberta Health Services Calgary, AB Canada

**Keywords:** osteoporosis, bone health, bone mineral density, fracture, survey, Facebook, advertisement, recruitment, women’s health, social media, bone, perspective

## Abstract

**Background:**

Surveys can help health researchers better understand the public’s perspectives and needs regarding prevalent conditions such as osteoporosis, which affects more than two-thirds of postmenopausal women. However, recruitment of large cohorts for survey research can be time-consuming and expensive. With 2.9 billion active users across the globe and reasonable advertising costs, Facebook (Meta Platforms, Inc) has emerged as an effective recruitment tool for surveys, although previous studies have targeted young populations (<50 years of age) and none have focused on bone health.

**Objective:**

We assessed the effectiveness and cost of using Facebook to recruit Canadian women aged ≥45 years to share their perspectives on bone health and osteoporosis via a web-based survey.

**Methods:**

We developed a 15-minute web-based survey with the goal of eliciting perspectives on bone health and osteoporosis. A Facebook advertisement was placed for 2 weeks in February 2022, during which time it was shown to women of age ≥45 years who resided in Canada, inviting them to participate and offering a chance to win 1 of 5 CAD $100 gift cards (at the time of this study [February 14, 2022], a currency exchange rate of CAD $1=US $0.79 was applicable). Those who clicked on the advertisement were taken to an eligibility screening question on the survey home screen. Individuals who confirmed eligibility were automatically directed to the first survey question. All individuals who answered the first survey question were considered participants and included in the analyses. We determined the survey reach, click rate, cooperation rate, completion rate, cost per click, and cost per participant. Sociodemographic characteristics of respondents were compared with data from the 2021 Canadian Census.

**Results:**

The Facebook advertisement was shown to 34,086 unique Facebook users, resulting in 2033 link clicks (click rate: 6.0%). A total of 1320 individuals completed the eligibility screening question, 1195 started the survey itself (cooperation rate: 58.8%), and 966 completed the survey (completion rate: 47.5%). The cost of the advertising campaign was CAD $280.12, resulting in a cost per click of CAD $0.14 and a cost per participant of CAD $0.23. The 1195 participants ranged in age from 45-89 years (mean 65, SD 7 years), 921 (93.7%) were of White ethnicity, 854 (88.3%) had completed some postsecondary education, and 637 (65.8%) resided in urban areas. Responses were received from residents of all 10 Canadian provinces and 2 of 3 territories. When compared to 2021 Canadian Census data, postsecondary education and rural residence were overrepresented in our study population.

**Conclusions:**

Facebook advertising is an efficient, effective, and inexpensive way of recruiting large samples of older women for participation in web-based surveys for health research. However, it is important to recognize that this modality is a form of convenience sampling and the benefits of Facebook recruitment must be balanced with its limitations, which include selection bias and coverage error.

## Introduction

Surveys are often used in health research to improve understanding of the public’s perspectives and needs regarding prevalent health conditions. However, survey studies often require large sample sizes and recruitment can be time-consuming, labor-intensive, and expensive, especially when traditional recruitment modes are used (ie, physical advertisements, mail-outs, and random digit dialing). Furthermore, traditional recruitment strategies do not always result in samples that are representative of the target population [1,2].

With more than 2.9 billion active users worldwide [3], Facebook (Meta Platforms, Inc) has emerged as an appealing tool for health research recruitment. The Facebook Ads Manager feature can be used to place advertisements that are shown to a large number of users at a relatively low cost and can be targeted to specific demographic and geographic groups (eg, gender, age, and country of residence). Several studies have described the effective recruitment of samples using Facebook, at a cost per participant that ranges from US $1.36 to $110.00 (average US $14-$17), depending on the target population and the nature of the research study [4,5]. Approximately 80% of Canadians are active Facebook users [6], and in a study describing the use of Facebook to recruit Canadian adults aged 35-74 years for a cross-sectional health survey, researchers were able to recruit a sample of participants with similar demographic characteristics to the general population at a cost per participant of CAD $2.18 (at the time of this study [February 14, 2022], a currency exchange rate of CAD $1=US $0.79 was applicable) [7].

Osteoporosis is a prevalent health condition, affecting more than two-thirds of postmenopausal women, with up to half experiencing a fragility fracture [8,9]. However, minimal data are available regarding the perspectives and preferences of Canadian women on the topics of bone health and osteoporosis prevention, limiting efforts to develop acceptable, preference-sensitive management strategies. Therefore, we sought to elicit the perspectives and preferences of Canadian women aged ≥45 years regarding bone health and osteoporosis via a web-based survey, using Facebook for recruitment. As the majority of existing literature describing Facebook recruitment has targeted younger populations (<50 years) and no studies, to our knowledge, have focused on bone health [4,5], a specific aim of our study was to assess the effectiveness and cost of using Facebook to recruit women aged ≥45 years to participate in a web-based survey on osteoporosis and to determine whether the demographic characteristics of our sample were similar to those of the Canadian population.

## Methods

### Study Design

We conducted a cross-sectional, self-administered electronic survey of Canadian women aged ≥45 years. Our primary aim was to elicit this population’s preferences and perspectives regarding bone health and osteoporosis (results to be published separately, awaiting ePub information for citation). This study describes a secondary aim of this study: to determine the effectiveness and cost of a Facebook-based recruitment strategy. To ensure transparency in the study design and recruitment process, we have followed the Consensus-Based Checklist for the Reporting of Survey Studies [10].

### Ethics Approval

This project was approved by the Conjoint Health Research Ethics Board at the University of Calgary (REB #20-1457).

### Survey Development

We developed an electronic survey that assessed the following parameters: sociodemographic characteristics, menopausal status, osteoporosis history, osteoporosis beliefs and preferences, perceived osteoporosis educational needs, and perceived osteoporosis research priorities. The survey was hosted on REDCap (Vanderbilt University) electronic data capture platform at the University of Calgary [11,12]. Pilot-testing was performed by members of a multidisciplinary osteoporosis management team (3 specialist physicians and an osteoporosis nurse) and a patient partner, each of whom provided feedback regarding face validity, content validity, ease of completion, and errors in branching logic. The final survey included 68 items, 34 of which were visible only if branching logic criteria were met. The survey included 11 screens: 1 screen (the home screen) provided an introduction to the study, a link to the informed consent form, and a single question to confirm eligibility; 7 screens contained survey questions; 1 screen provided participants with the option to enter into a draw for a gift card; 1 screen provided the option to be contacted by the research team for future studies; and 1 screen contained a thank you message). Pilot-testing indicated that the survey required <15 minutes to complete.

### Recruitment

**Figure 1:**
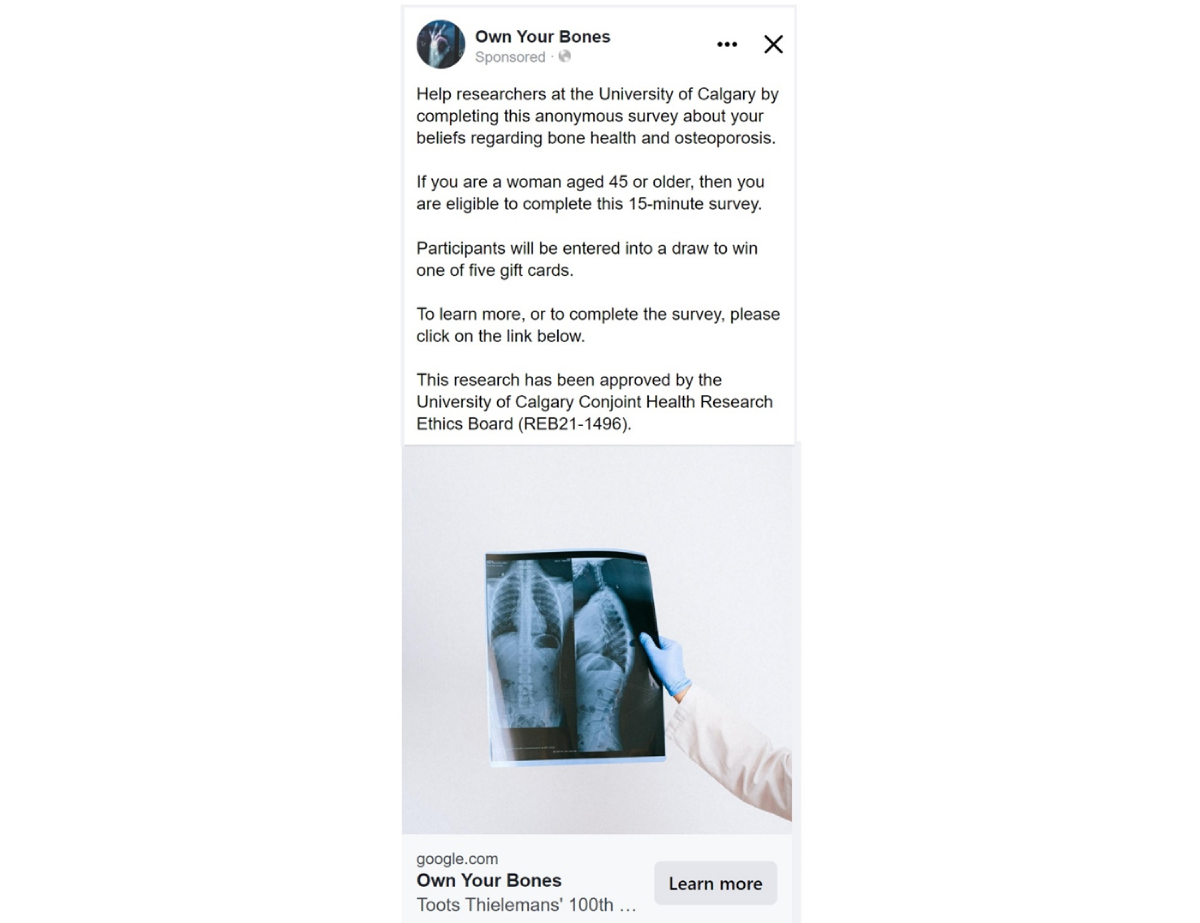
Facebook advertisement.

Using Facebook Ads Manager, an advertisement (shown in Figure 1) was created to provide basic details about the study and included a public link to the survey. We also created a Facebook page that included a description of the research project and offered visitors the opportunity to interact and ask questions of the research team. Creation of the Facebook page was straightforward, taking approximately 2 hours. Publishing the page required 2 images to be uploaded (profile and cover photos); freely available stock images were used for this purpose. A 14-day advertising campaign was launched in February 2022, during which time the advertisement was shown to individuals from the following prespecified target demographic: women aged ≥45 years who resided in Canada. The advertising budget was prespecified at CAD $20 per day (CAD $280 for 14 days). No other recruitment strategies were used.

Individuals who clicked on the advertisement were taken to the survey launch page, which contained a link to an implied consent form and an eligibility screening question. Providing an answer to the eligibility screening question (“Are you a woman aged 45 years or older?”) was taken as implied consent to participate. Individuals who responded “No” to this question were not given the option to continue the survey. Those who responded “Yes” were automatically taken to the first survey question and invited to start the survey. All individuals who answered the first survey question were classified as survey participants.

### Data Collection

All survey participants received an automatically generated ID number in the REDCap system, allowing the capture of both complete and incomplete survey responses. Survey questions were divided into the following six sections, each presented on a separate screen: (1) age and menopausal status (5 items); (2) health history (13 items); (3) perspectives regarding bone health and medication use (21 items, presented on 2 separate screens); (4) perceived priorities for bone health research (11 items); (5) perceived bone health education needs (7 items); and (6) demographics (10 items). Participants were required to provide an answer to every question on each screen before proceeding to the next screen. For questions pertaining to medical history and personal preferences, respondents had the option of answering “not sure.” For demographic questions, one of the response options was “prefer not to answer.” Participants were not able to return to previous screens and change their answers, although they could exit the survey at any time.

We did not use item randomization or adaptive testing, and our survey was not set up to check for multiple responses from the same participant (ie, multiple responses from the same IP address).

Data from the 2021 Canadian Census were obtained from the Statistics Canada 2021 Canadian Census data tables [13] with the view of comparing the study cohort demographics with those of the Canadian population.

### Data Analysis

The demographic characteristics of participants were examined using descriptive statistics; means and SDs were calculated for continuous variables and percentages were calculated for categorical variables. All available responses to each survey question were analyzed (ie, analyses were not restricted to survey completers). We evaluated the following measures of recruitment effectiveness and cost: click rate (number of clicks on the advertisement or number of users the advertisement was shown to), cooperation rate (number of participants with complete or partial responses/[number of participants with complete or partial responses + number who clicked on the advertisement but did not participate]), completion rate (number of participants with complete responses/[number of participants with complete or partial responses + number who clicked on the advertisement but did not participate]), cost per click (total cost or number of clicks on advertisement), cost per participant (total cost or number of participants), and cost per completer (total cost or number of completers). Where applicable, we used calculations recommended by the American Association for Public Opinion Research (AAPOR), keeping in mind the limitations of these calculations for nonprobability samples [14]. Our cooperation rate calculations reflected AAPOR calculations for “Cooperation Rate 4,” and our completion rate calculations reflected AAPOR calculations for “Cooperation Rate 3.”

## Results

**Figure 2:**
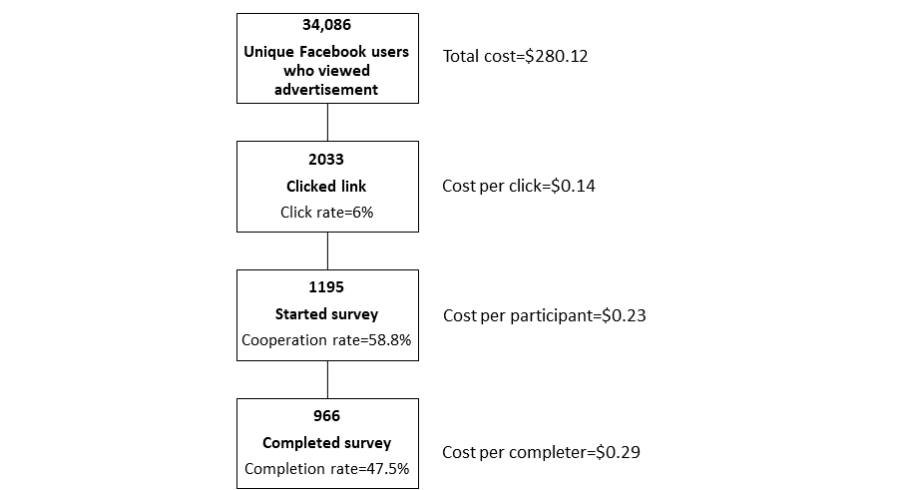
Results of a 14-day Facebook advertising campaign to recruit women aged ≥45 years to complete an electronic survey regarding their perspectives on bone health and osteoporosis. Costs are presented in CAD (at the time of the study, a currency exchange rate of CAD $1=US $0.79 was applicable).

The Facebook advertising campaign ran from February 3 to 18, 2022, during which time the advertisement was shown to 34,086 unique users (2435 per day). As shown in Figure 2, this generated 2033 link clicks, resulting in a click rate of 6%. A total of 1320 individuals completed the eligibility screening question, of which 1291 (97.8%) were eligible to participate. Of the 2033 individuals who clicked the link, 1195 started the survey and were considered participants (cooperation rate: 58.8%) and 966 completed the survey (completion rate: 47.5%). Of the 229 participants who started but did not complete the survey, 16 stopped at the age and menopausal status section, 101 at the health history section, 52 at the perspectives regarding bone health and medication use section, 36 at the perceived priorities for bone health research and education sections, and 24 at the demographics section. The total cost of the advertising campaign was CAD $280.12, which corresponded to a cost per click of CAD $0.14, a cost per participant of CAD $0.23, and a cost per completer of CAD $0.29. Throughout the advertising campaign, the study team monitored the Facebook page and provided answers to questions posed by page visitors. This required approximately 4 hours of principal investigator time and 4 hours of research coordinator time over the 14-day period.

Demographic characteristics of participants are shown in Table 1. All participants indicated on the eligibility screening question that they were women aged ≥45 years and therefore eligible to participate. In a later survey question that asked about gender identity, 1193 (99.7%) of respondents indicated that they best identified as a woman, 2 (0.2%) as a man, and 1 (0.1%) as a 2-spirit. We did not exclude participants from the analysis on the basis of gender identity. Participants ranged in age from 45 to 89 years, (mean 65, SD 7 years), 921 (93.7%) were of White ethnicity, 854 (88.3%) had completed some form of postsecondary education, and 637 (65.8%) resided in urban areas. All 10 Canadian provinces and 2 of 3 territories were represented, with 256 (26.5%) participants residing in Ontario and 240 (24.8%) in Alberta.

We used data from the 2021 Canadian Census [13] to evaluate whether our sample was demographically similar to the Canadian population. Census data were available for Canadian women aged ≥45 years residing in 3 provinces (British Columbia, Alberta, and Ontario) for 3 sociodemographic characteristics: marital status, employment status, and educational attainment (summarized in Table 2). We were unable to formally compare characteristics between our study population and the census population due to differences in how responses were categorized. However, similar proportions of our study population and the census population were married (624/968 [64.5%] and 3,111,855/5,537,435 [56.2%], respectively). The proportion of individuals living with common-law partners, who were separated or divorced, widowed, and single were also comparable between our study population and the census population. Regarding employment status, 230/968 (23.7%) of the study population was currently working full- or part-time, whereas 2,971,020/5,367,825 (55.3%) of the census population reported having an employment income. With respect to educational attainment, 854/968 (88.3%) of the study population had some postsecondary education, while 3,190,155/5,572,720 (57.2%) of the census population had a postsecondary certificate or degree. Census data regarding the area of residence were available for women and men of all ages from 3 provinces (British Columbia, Alberta, and Ontario). Among the study population, 324/967 (33.5%) resided rurally, compared to 1,347,823/9,913,614 (13.6%) from the census population. Comparable 2021 census data were not available for ethnicity province of residence or household income.

**Table 1:** Demographic characteristics of survey participants (N=1195).

Characteristic		Values, n (%)
**Age (years) (n=1195)**		
	45-54	104 (8.7)
	55-64	407 (34.1)
	65-74	568 (47.5)
	≥75	116 (9.7)
**Ethnicity (n=968)**		
	East or Southeast Asian	10 (1.0)
	Indigenous	21 (2.1)
	Latino	1 (0.1)
	Black	0 (0.0)
	Middle Eastern	6 (0.6)
	South Asian	4 (0.4)
	White	921 (93.7)
	Other	11 (1.1)
	Do not know	2 (0.1)
	Prefer not to answer	7 (0.7)
	Total visible minority	42 (4.3)
**Educational attainment (n=968)**		
	Some or completed elementary or high school	86 (8.8)
	Some or completed trade, community college, or university program	707 (73.1)
	Advanced degree (MSc, PhD, MD, etc)	147 (15.2)
	Other or prefer not to say	29 (2.9)
	Total with some or completed postsecondary education	854 (88.3)
**Employment status (n=968)**		
	Full time	156 (16.1)
	Part time	74 (7.6)
	Not working or on benefits	26 (2.7)
	Not working or seeking work	12 (1.2)
	Retired	649 (67.0)
	Homemaker	18 (1.9)
	Other	25 (2.6)
	Prefer not to say	8 (0.8)
**Gross household income (CAD)^a^ (n=967)**		
	CAD $0-$49,999	194 (20.1)
	CAD $50,000-$99,999	268 (27.7)
	≥CAD $100,000	205 (21.2)
	Prefer not to say	300 (31.0)
**Marital status (n=968)**		
	Single	56 (5.8)
	Married	624 (64.5)
	Common law	62 (6.4)
	Living with partner	9 (0.9)
	Separated	19 (2.0)
	Divorced	93 (9.6)
	Widowed	90 (9.3)
	Prefer not to say	15 (1.5)
**Area of residence (n=968)**		
	Urban	372 (38.4)
	Suburban	265 (27.4)
	Rural	324 (33.5)
	Prefer not to say	7 (0.7)
	Total in urban or suburban areas	637 (65.8)
**Province or territory of residence (n=967)**		
	British Columbia	169 (17.5)
	Alberta	240 (24.8)
	Saskatchewan	73 (7.5)
	Manitoba	35 (3.6)
	Ontario	256 (26.5)
	Quebec	45 (4.7)
	New Brunswick	29 (3.0)
	Newfoundland and Labrador	29 (3.0)
	Nova Scotia	69 (7.1)
	Prince Edward Island	13 (1.3)
	Yukon	8 (0.8)
	Northwest Territories	0 (0.0)
	Nunavut	1 (0.1)

^a^At the time of this study (February 14, 2022), a currency exchange rate of CAD $1=US $0.79 was applicable.

**Table 2:** Data from the 2021 Canadian Census demonstrating demographic characteristics of Canadiana women aged ≥45 years.

Characteristic			Values, n (%)
**Age (years) (n=8,844,010)**			
	45-54	2,381,670 (26.9)	
	55-64	2,665,600 (30.1)	
	65-74	2,113,405 (23.9)	
	≥75	1,683,335 (19.0)	
**Marital status (n=5,537,435)**			
	Married	3,111,855 (56.2)	
	Living common law	335,960 (6.1)	
	Never married and not living common law	453,895 (8.2)	
	Separated	208,500 (3.8)	
	Divorced	626,680 (11.3)	
	Widowed	800,545 (14.5)	
**Employment status (n=** **5,367,825)**			
	With employment income	2,971,020 (55.3)	
	Without employment income	2,396,810 (44.7)	
**Educational attainment (n=** **5,572,720)**			
	No certificate, diploma, or degree	829,380 (14.9)	
	High school diploma or equivalent	1,553,185 (27.8)	
	Apprenticeship, trades certificate, or diploma	404,980 (7.3)	
	College, nonuniversity, or university below bachelor’s	1,434,790 (25.7)	
	Bachelor’s degree	886,515 (15.9)	
	University certificate, diploma, or degree above bachelor’s	463,870 (8.3)	

## Discussion

### Principal Results

This is the first study, to our knowledge, to describe the effectiveness and cost of using Facebook to recruit older women (age range 45-89 years) to participate in a web-based survey pertaining to bone health. Over the course of a 14-day Facebook advertising campaign, we recruited 1195 Canadian women aged ≥45 years to participate in a 15-minute web-based survey about bone health and osteoporosis. With a cooperation rate of 58.5% (1195/2033) and cost per participant of CAD $0.23, our findings suggest that Facebook may be even more effective for recruiting older (ie, age ≥45 years) women for participation in research than what has been reported in younger populations [4,5]. Compared to the general population, survey participants were more likely to have completed postsecondary education and more likely to live in a rural area, suggestive of selection bias.

### Limitations

Our findings should be considered in the context of several limitations. First, the use of Facebook as the sole mode of recruitment for this survey was a form of nonprobability sampling and, as such, did introduce the potential for selection bias and coverage error. Approximately 20% of the Canadian population does not use Facebook [6], and furthermore, beyond setting a target budget and target demographics for the Facebook advertisement, the research team was not able to control or monitor who the advertisement was shown to, although we hypothesize that it would have been more likely to be shown to those who spend the most time on the platform. Direct comparison of our study cohort with 2021 Canadian Census data was not possible, but the available data do suggest the presence of selection bias. That is, those with postsecondary education and those residing rurally were overrepresented in our study cohort. Additionally, we did not use quality checks, such as questions designed to test participants’ attention, nor were we able to measure the time taken for each participant to complete the survey. Therefore, we cannot rule out the possibility that some participants did not take the time to read and answer each question thoughtfully. A further limitation is that we did not track IP addresses, so it was not possible to determine if any of the participants completed the survey more than once or used automated bots to increase the probability of winning a gift card, as has been reported in other studies [15]. Reassuringly, among the 669 respondents who entered the gift card draw, we did not identify any duplicate names or contact information.

### Comparison With Prior Work

The use of a Facebook recruitment strategy for our study was both more effective and less expensive than anticipated, based on what has been reported in the existing literature. For example, our cooperation rate was higher than the rates of 20%-40% reported by 2 recent studies using Facebook to recruit for web-based health research surveys [16,17]. This may be explained by the provision of an incentive (a chance to win 1 of 5 CAD $100 gift cards), although not all studies have found that incentives increase recruitment [5]. Other factors that may have motivated viewers to click on our advertisement (Figure 1) included the image, which increased visual appeal, and the inclusion of the name of the research institution and the ethics approval number, both of which demonstrated legitimacy. Studies targeting women may also be more conducive to Facebook recruitment, as women tend to be overrepresented in Facebook advertising campaigns [4] and are more likely to engage with advertisements for health research participation [7]. The low cost per participant observed in our study (CAD $0.23, compared to an average of US $15 in other studies [4,5]) may be explained by the low participant burden (ie, a 15-min electronic survey). In a recent study by Walsh and Carter-Bawa [16], where Facebook was used to recruit older adults for a web-based survey on lung cancer screening, the cost per study completer was $3.14, compared to CAD $0.29 in our study. This cost discrepancy may reflect the use of quality and integrity checks in Walsh and Carter-Bawa’s [16] study. Shaver et al [7] used Facebook to recruit 1048 adults aged 35-74 years residing in Newfoundland and Labrador for a cross-sectional web-based health survey. Their click rate (2067/34,012) was 6%, response rate (similar to cooperation rate; 1048/2067) was 50%, and cost per participant was CAD $2.18. We hypothesize that their higher cost per participant was because it was more expensive for them to show the advertisement to their target number of unique Facebook users, possibly because their study involved a much smaller target population than ours (~525,000 individuals residing in Newfoundland and Labrador). This suggests that Facebook recruitment might be more economical when the target population is larger.

Most existing studies indicate that Facebook can be used to recruit samples with similar demographics to the general population, although individuals of White ethnicity and those who have completed postsecondary education are often overrepresented [4,5]. Although no appropriate Canadian Census data were available for ethnicity, we found that individuals with postsecondary education were overrepresented in our sample, as were those residing rurally. Our findings suggest that Facebook may be an effective strategy to recruit rural populations, which are typically hard to reach, for participation in web-based surveys.

### Conclusions

Facebook advertising is an efficient, inexpensive way to recruit participants for cross-sectional web-based surveys and appears to be effective in older populations. Researchers may wish to use Facebook for recruitment when looking to quickly obtain a large number (ie, >1000) of survey responses from a large cohort, across a broad geographical region, at a low cost. However, it is important to recognize that Facebook recruitment is a form of convenience sampling and the benefits of this modality must be balanced with its limitations, such as the introduction of selection bias and coverage error.

## Abbreviations

AAPOR: American Association for Public Opinion Research

## Data Availability

The data sets generated and analyzed during this study are available from the corresponding author on reasonable request.
